# Pancreatic Cancer with Mutation in BRCA1/2, MLH1, and APC Genes: Phenotype Correlation and Detection of a Novel Germline BRCA2 Mutation

**DOI:** 10.3390/genes13020321

**Published:** 2022-02-09

**Authors:** Maria Teresa Vietri, Giovanna D’Elia, Gemma Caliendo, Luisa Albanese, Giuseppe Signoriello, Claudio Napoli, Anna Maria Molinari

**Affiliations:** 1 Department of Precision Medicine, University of Campania “Luigi Vanvitelli”, 80138 Naples, Italy; annamaria.molinari@unicampania.it; 2 Unity of Clinical and Molecular Pathology, AOU, University of Campania “Luigi Vanvitelli”, 80138 Naples, Italy; giovanna.delia@policliniconapoli.it (G.D.); gemma.caliendo@unicampania.it (G.C.); luisa.albanese@studenti.unicampania.it (L.A.); 3 Statistical Unit, Department of Precision Medicine, University of Campania “Luigi Vanvitelli”, 80138 Naples, Italy; giuseppe.signoriello@unicampania.it; 4 Department of Advanced Medical and Surgical Sciences (DAMSS), University of Campania “Luigi Vanvitelli”, 80138 Naples, Italy; direzione.immunoematologia@unicampania.it; 5 Clinical Department of Internal Medicine and Specialistic Units, AOU, University of Campania “Luigi Vanvitelli”, 80138 Naples, Italy

**Keywords:** pancreatic ductal adenocarcinoma, hereditary breast and ovarian cancer syndrome, hereditary nonpolyposis colon cancer syndrome, familial adenomatous polyposis, BRCA genes, MMR genes

## Abstract

Pancreatic ductal adenocarcinoma (PDAC) is the seventh leading cause of cancer death worldwide; most of cases are sporadic, however about 5% to 10% report a hereditary predisposition. Several hereditary syndromes have been associated with familial pancreatic cancer (FPC) onset, including hereditary breast and ovarian cancer syndrome (HBOC), Lynch syndrome (LS), Familial atypical multiple mole melanoma (FAMMM), Familial adenomatous polyposis (FAP), Li–Fraumeni syndrome (LFS), Peutz–Jeghers syndrome (PJS), and Hereditary pancreatitis (HP).The aim of this study was to determine the mutational status of a cohort of 56 HBOC families, 7 LS families, 3 FAP and FAMMM families, and 1 LFS family with at least one case of PDAC. Mutation analysis of *BRCA1/2, ATM, CHEK2, PALB2, RAD51C, RAD51D, NBN, CDH1, TP53, MLH1, MSH2, MSH6,* and *PMS2* genes, showedmutation in *BRCA1/2, MLH1*, and *APC* genes. We founda high mutation rate in patients belong HBOC and LS families, with a percentage of 28.6% in both syndromes and prevalence in HBOC of *BRCA2* mutations with one case of double mutation in *BRCA2* gene. In FAP family, we found a pathogenic mutation in *APC* gene in 1/3 families. We observed an early onset of PDAC and a lower survival in PDAC patients belonging to mutated families, while no evidence of possible pancreatic cancer cluster regions was found. Moreover, we identified a novel *BRCA2* germline mutation, c.5511delT (p.Phe1837LeufsX3), not reported in any database, that segregated with disease in HBOC patients. Mutational analysis was extended to family membersof mutated patients, both healthy and cancer affected, which revealed 23 unaffected family members that inherited the proband’s mutation. Although correlative by its nature, the presence of a *BRCA* mutation in PDAC patients may have benefits in terms of optimized treatment and longer outcome.

## 1. Introduction

Pancreatic ductal adenocarcinoma (PDAC) is the twelfthmost common tumor and the seventh main cause of cancer death worldwide [1]. It represents about 5% of all malignancy cases, with the overall survival (OS) 5 years worse than other cancers [2]. Recently, a 2.3-fold increase has been seen in the global number of cases and deaths from PDAC, with a 3 times higher incidence in most developed countries [3]. Most of the PDAC cases are sporadic, but about 5% to 10% report a hereditary predisposition [4].

Pancreatic cancer arises in several hereditary syndromes, including Hereditary breast and ovarian cancer (HBOC), Lynch syndrome (LS), Familial atypical multiple mole melanoma (FAMMM), Familial adenomatous polyposis (FAP), Li–Fraumeni syndrome (LFS), Peutz–Jeghers syndrome (PJS), and Hereditary pancreatitis (HP) [5,6,7,8]. Most familial pancreatic cancer (FPC) is attributable to HBOC syndrome that results from germline mutations in *BRCA1/2* genes and other genes such as *ATM, BRIP1, CHEK2, RAD50*, and *RAD51C* [9,10]. In FPC belonging to HBOC, *BRCA2* mutations are the most common genetic alteration, observed in about 5–10% of cases [11].

It was shown that about half of the patients with PDAC report pathogenic germline variants, even if they do not have a family history of pancreatic carcinoma. Moreover, the mutation presence could have therapeutic implications [12]. The American Society of Clinical Oncology (ASCO) and National Comprehensive Cancer Network (NCCN) guidelines to date recommend universal germline tests for all patients with PDAC [13] in order to identify germline mutation in patients that may benefit from alternative treatments. Moreover, identification of germline mutations in a patient will allow for testing family members at risk ofthe same or other associated cancers.

Immunetherapies or molecularly targeted agents have been recently introduced into cancer clinical practice [14,15,16], but it is still unclear whether they promote benefits in PDAC patients. For patients with *BRCA* mutation, positive results data have been shown in clinical trials on poly (ADP-ribose) polymerase (PARP) inhibitors, as in use of anti-PD-1 antibodies for patients with mismatch repair (MMR) mutation [17]. In a retrospective analysis, patients with PDAC, with a *BRCA* mutation, after treatment with the combination regimen of leucovorin calcium, fluorouracil, irinotecan hydrochloride, and oxaliplatin (FOLFIRINOX), had a longer OS than those without the mutations [18]. No differences in survival outcomes were observed in patients treated with Gemcitabine/nab-paclitaxel and FOLFIRINOX, so alternative treatments could be considered in patients with PDAC [19].

The aim of the study was to identify germline mutations in 14 susceptibility genes in HBOC, LS, FAP, FAMMM, and LFS Italian families with at least one case of pancreatic cancer, correlating with phenotypic characteristics of FPC families. Moreover, we describe a novel *BRCA2* germline mutation not reported in any database, in a patient and her family. Finally, we carried out a literature analysis to ongoing trials on the treatment of mutated patients.

## 2. Materials and Methods

### 2.1. Patients

This study was carried out in accordance with the World Medical Association Helsinki Declaration, adopted in 1964 and amended in 1975, 1983, 1989, 1996, and 2000. Informed consent was obtained from all subjects, and the study was approved and conducted according to the ethical guidelines of the University of Campania “Luigi Vanvitelli” (n.469-23/07/2019).In this study, we enrolled 56 HBOC families, 7 LS families, 3 FAP families, 2 FAMMM families, and 1 LFS family with at least one case of pancreatic cancer. The probands were affected with pancreatic, colorectal, breast or ovarian cancer, pr melanoma and sarcoma (Table 1), with an age interval of 26–85 years.

In 56 HBOC families, 64 cases of PDAC recur (25 females and 39 males) with mean age of onset of 66 years. In the 7 LS families, 8 cases of PDAC occur (3 females and 5 males), the mean age of onset is 58.6 years. In 3 FAP families, 5 cases of PDAC recur (2 females and 3 males), with a mean age of 48.8 years. In 2 FAMMM families, 2 cases of PDAC occur (1 female and 1 male), the mean age of onset is 62 years, while in the LFS family, there was 1 case of male PDAC, at 65 years (Table 1).

All selected patients received genetic counselling. The case and family history were collected, and the pedigree was generated. Mutational analysis was extended to family members of mutated patients.

### 2.2. Mutational Analysis

Peripheral blood sample were collected from all patients. Genomic DNA was extracted using the Wizard Genomic DNA purification kit (Promega Corporation, Madison, WI, USA) according to the manufacturer’s instructions.

For mutational analysis, we used the TruSight Sequencing Cancer Panel on a MiSeq platform (Illumina) that analyzes 14 genes, *BRCA1(NM_007295), BRCA2(NM_000059), ATM (NM_000051.4), CHEK2(NM_007194),PALB2(NM_024675), RAD51C(NM_058216), RAD51D(NM_002878), NBN(NM_002485), CDH1 (NM_004360), TP53 (NM 000546), MLH1 (NM 000249), MSH2 (NM 000251), MSH6 (NM 000179.3), * and *PMS2 (000535.7).*

The presence of the point mutation was confirmed on the other blood sample by Sanger sequencing, as previously described [20]. The results were analyzed using Mutation Surveyor^®^ software, version 3.24 (Softgenetics, State College, PA, USA). Molecular analysis in family members of mutated probands was performed by Sanger sequencing, as previously described [21,22].

### 2.3. Statistical Analysis

Continuous variables were summarized as mean and standard deviation, and categorical variables as absolute and relative frequencies. Categorical variables were compared by Chi-Square test. Significance was assumed for *p*-values less than 0.05.

## 3. Results

We found pathogenic mutations in 28.6% (16/56) HBOC proband, 28.6% (2/7) LS, and 33.3% (1/3) FAP, while we did not findmutations in patients belonging to FAMMM and LFS families. Mutations identified occur in *BRCA1, BRCA2*, *MLH1*, and *APC* genes, while in other investigated genes no mutations were observed. Eighty PDAC cases recur in families with HBOC, LS, FAP, FAMMM, and LFS syndromes (Table 1).

PDAC patients are predominantly male, even if no statistically significant gender differences are observed (Table 2).

Table 2 shows age of onset and death of PDAC patients. We observe a lower survival in mutated patients (1, 2 years) than in non-mutated patients (2, 3 years), although no statistically significant differences are found between two groups.

### 3.1. HBOC Families

Out of 56 HBOC probands, 16 (28.6%) reported a pathogenic mutation, particularly 6/56 (10.7%) in *BRCA1*, 9/56 (16.1%) in *BRCA2*, and 1/56 (1.8%) reported a double mutation (DM), both in *BRCA2*.

**Figure 1:**
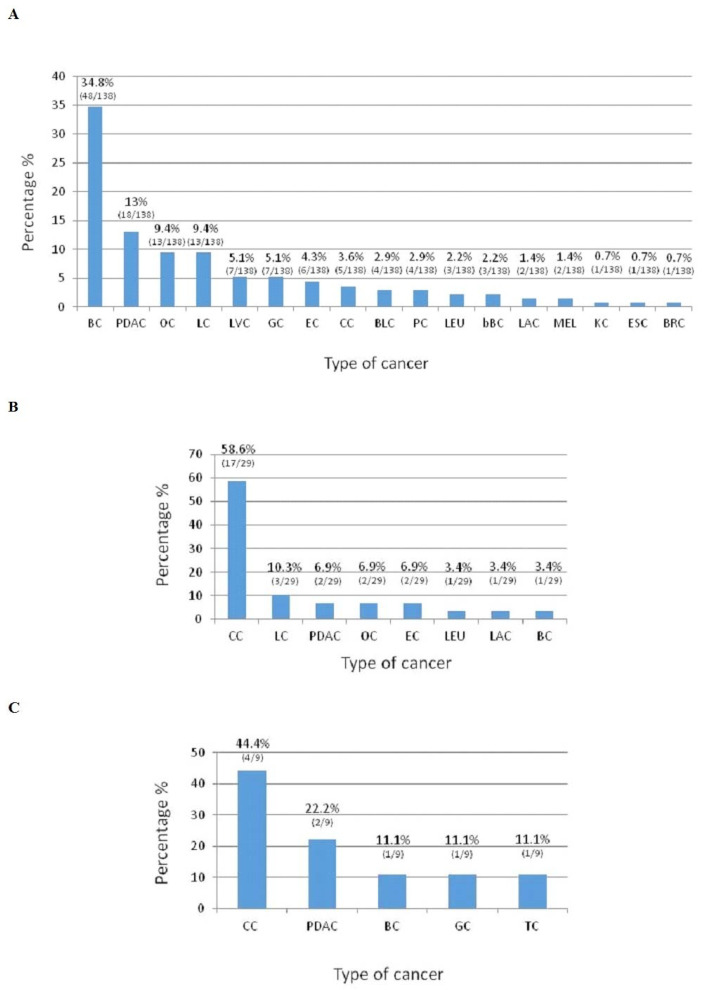
Cancer types that occur in HBOC mutated families (**A**). Cancer types that occur in LS mutated families (**B**). Cancer types that occur in FAP mutated family(**C**). BC: Breast cancer; PDAC: Pancreatic ductal adenocarcinoma; OC: Ovarian cancer; LC: Lung cancer; LVC: Liver cancer; GC: Gastric cancer; EC: Endometrial cancer; CC: Colon cancer; BLC: Bladder cancer; PC: Prostate cancer; LEU: Leukemia; bBC: Bilateral Breast cancer; LAC: Laryngeal cancer; MEL: Melanoma; KC: Kidney cancer; ESC: Esophageal cancer; BRC: Brain cancer; and TC: Thyroid cancer.

The number and percentage of cancers type that occur in mutated HBOC families are reported in Figure 1A; PDAC is the second recurrent cancer, resulting in 13% of cases. Table 3 shows the frequency of cancer types that occur in mutated and non-mutated HBOC families. A statistically significant difference is observed in OC onset.

**Figure 2:**
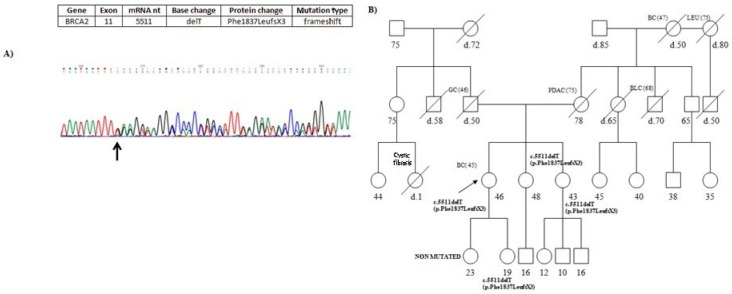
(**A**) Partial electropherogram of *BRCA2* exon 11. The novel germline mutation c.5511delT (p.Phe1837LeufsX3) results in chain termination at codon 1840. (**B**) Pedigree of the proband carrying the *BRCA2* novel mutation c.5511delT (p.Phe1837LeufsX3). The ages at diagnosis are indicated in brackets. Her mother died at 80 years old, was affected with PDAC.

One of the *BRCA2* mutations, specifically c.5511delT (p.Phe1837LeufsX3), was a novel germline mutation, not previously reported in any database, previously observedas somatic mutation in tissue of mucoepidermoid carcinoma [23]. This mutation, localized in exon 11, consists of a thymine deletion at nucleotide 5511, which results in the introduction of a premature stop codon at position 1840 (Figure 2A). It was identified in a 46-year-old woman affected with breast cancer diagnosed at the age of 45. Figure 2B reports the pedigree of the proband; her mother was affected with pancreatic cancer. Moreover, the analysis was extended in the sisters of 48 and 43 years, that carried the mutation. Finally, the analysis was conducted to daughters of 23 and 19 years and it revealed the presence of mutation only in the daughter of 19 years.

**Figure 3:**
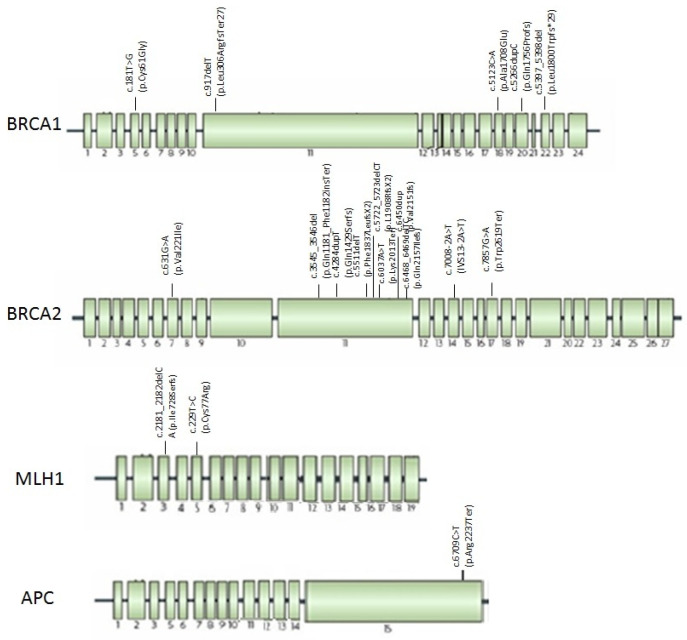
Mutations found in HBOC, LS, and FAP families with PDAC localized trough *BRCA1, BRCA2, MLH1*, and *APC* genes.

Figure 3 shows the location through *BRCA1/2* genes of the mutations found. The mutations are located throughout the length of gene, in *BRCA2* most of them fall in exon 11.

Mutational analysis was extended to 60 family members of 16 mutated patients, both healthy and cancer affected. The results of genetic test were summarized in Table 4. Twenty-sixfamily members resulted wild-type and 34 inherited the proband’s mutation, of these 11 with cancer and 23 unaffected.

### 3.2. LS Families

Out of sevenprobands, two (28.6%) reported a pathogenic mutation in *MLH1* gene. The mean age of pancreatic cancer onset was 62.5 years in mutated patients and 57.3 years in non-mutated patients.

The number and percentage of cancers type that occur in LS mutated families were reported in Figure 1B. PDAC is the third recurrent cancer and happen in 6.9% of cases.

The mutations found were in exon 3 and 5 of *MLH1* gene, as shown in Figure 3.

Mutational analysis was extended to three family members of one mutated patient, both healthy and cancer affected; in the second family, no family member was available for genetic testing. The results of genetic test were summarized in Table 4; no family member inherited the mutation.

### 3.3. FAP Families

Out of three probands, one (33.3%) reported a pathogenic mutation in *APC* gene. The mean age of pancreatic cancer onset was 47.5 years in mutated patient and 49.6 years in non-mutated patients.

The number and percentage of cancers type that occur in *APC* mutated family were reported in Figure 1C. PDAC is the second recurrent cancer and occurred in 44.4% of cases.

The mutation found was in exon 15 of *APC* gene, as showed in Figure 3.

Mutational analysis was extended to six family members, both healthy and cancer affected, of mutated patient; four inherited the proband’s mutation (Table 4).

## 4. Discussion

The main finding of the present study is that FPC occurs in both males and females at a similar rate, in HBOC LS, FAP, and FAMMM, reporting an earlier onset, with a mean age of 62.8 in mutated patients and 64.5 in non-mutated patients (Table 2); although no statistically significant differences are found between mutated and non-mutated patients, we observe a lower survival in PDAC patients belonging to families with mutation, hypothesizing that the mutation presence could be correlated to a worse phenotype, in terms of OS. FPC incidence is higher in men than in women [1]; moreover, FPC shows a trend toward a younger onset of 58–68 years, compared to 61–74 years in sporadic [24,25]. These data could be supported from a greater number of analyzed cases.

Moreover, the mutation rate in HBOC and LS, was higher (28.6%) than data previously found, showing the frequency of mutation in *BRCA1/2* genes be up to 17% in HBOC and in MMR genes up to 12% in LS [26]. The difference in mutation prevalence could be influenced by the selection of the patients included. Studies conducted in FPC patients report a lower mutation rate than a study that included FPC families [27,28].

We observe that the BRCA2 germline mutation is associated with FPC relative risk of 3.5 to 10 as compared to non-carriers; while the relative risk of FPC developing in *BRCA1* mutation carriers is approximately 2.26 to 3% [29,30]. As shown in Figure 1A, in HBOC mutated families, PDAC is the second recurrent cancer and happen in 13% of cases, higher than OC. Interestingly, by comparing the prevalence of cancers that recur in mutated and non-mutated HBOC families, statistically significant difference is observed in OC onset (Table 3), confirming that OC patients exhibit a high mutational rate [31]. Patients with *BRCA2* mutations are at increased risk for PDAC onset; it has been reported that loss of *BRCA2* function predisposes the pancreas to profound DNA damage that determines a major frequency of invasive neoplasia. In addition, multiple cases of PDAC are found in some large *BRCA2* mutated families compared to families with mutations in other genes [32]. The *BRCA2* gene was the most commonly mutated in FPC; we found BRCA2 mutations in 16.1% of patients, value included in range that reports BRCA2 mutations in 3.7–17.2% of FPC patients [27,33,34]. *BRCA1* mutations in FPC have not been reported as well as *BRCA2*, but with a lower frequency [35]. Here, we found a higher percentage of *BRCA1* mutations in 10.7% of patients, not in line with data reported (1.2–2.6%) [26,27,36]. Moreover, in a retrospective study on FPC families, *BRCA1* mutations occur in 9.5% [28], this could be due to a different ethnic distribution.

In addition, one patient (1.8%) showed DM in *BRCA2*. The family with DM does not show a more severe phenotype than families with a single mutation. The *BRCA2* DM c.631G>A (p.Val211Ile)/c.7008-2A>T (IVS13-2A>T), was described in unrelated HBOC families [37] and was previously reported in a patient with PDAC [38]. Moreover, it was previously reported in male breast cancer patients [39,40], suggesting high penetrance in PDAC and MBC development in carriers of DM c.631G>A (p.Val211Ile)/c.7008-2A>T (IVS13-2A>T).Remarkably, in the present study, we describe a novel germline mutation in *BRCA2* gene, c.5511delT (p.Phe1837LeufsX3), previously observed in tissue of mucoepidermoid carcinoma [23]. We identified this mutation in a woman affected with breast cancer; her mother was affected with PDAC and died at 78 years old. The mutation is very likely inherited from the maternal side, in which occurs HBOC related tumors (Figure 2B).

In *BRCA1/2*, an ovarian cluster region (OCCR) was reported; in *BRCA1* gene OCCR is located between c.1380–4062, and in *BRCA2* between c.3249–5681 and c.5645–7471 [41]. Previously, Toss et al. [42] observed two possible pancreatic cancer cluster regions in *BRCA1* and *BRCA2* genes. The *BRCA1* region is located between c.3239–c.3917 (exon 10) and *BRCA2* region within c.7180–c.8248 (exon 14–18). Only one of our identified mutations, *BRCA2* c.7857G>A (p.Trp2619Ter), falls in exon 17, in BRCA pancreatic cluster region reported. In our family *BRCA1/2* mutations are located throughout the gene, although most of BRCA2 mutations fall between codons 3545–6469, in exon 11 (Figure 3), where many mutations were found in HBOC patients. This suggests no evidence of possible pancreatic cancer cluster regions in *BRCA* genes, which could influence the PDAC risk, however a larger number of studies is necessary to clarify this association.

We show pathogenic mutation in *MLH1* gene in 28.6% of patients among LS families. MMR mutations were reported in a large range, between 1–12.5%, of FPC cases, with major frequency in *MSH6* gene, followed by *MSH2* and *MLH1* genes [4,28,35,43]. Additionally, in this case, the difference could reflect the choice to enroll FPC families in our study. Previous studies that included FPC patients reported a lower mutation rate, such as the study by Yurgelun M et al. which reported mutations in 1–1.3% of cases, while studies that enrolled FPC families showed higher mutation rates (11.1–12.5%) [28,35].

In LS mutated families, PDAC is the third recurrent cancer and occur in 6.9% of cases, together with EC and OC (Figure 1B). The revised Bethesda Guidelines (RBG) indicate PDAC as one of the LS-associated cancers [44]. It was shown that MMR mutations increased PDAC risk by 8.6-fold and cumulative PDAC risk was 3.7% [17]. A recent study found that PDAC risk was 7.8 in *MLH1* mutated patients [45]. In our patients, we found mutations in *MLH1* but not in other MMR genes. Until now, no pancreatic cancer cluster regions were reported in *MLH1* gene or in other MMR genes. The two *MLH1* mutations found were in exons 3 and 5 of gene (Figure 3); therefore, an indication of no possible pancreatic cancer cluster regions can also be suggested given the small number of mutations found.

In FAP families, 33.3% of patients showed a pathogenic mutation in *APC* gene. Pancreatic cancer onset in FAP patients is rare, representing about 1% of extracolonic malignancies [46]; today, few germline *APC* mutations have been found in FPC patients. In one study, it 1.4% of FPC patients with *APC* mutations were observed [43]; another study reported the presence of double mutation in *APC* and *BRCA2* genes in a patient with FPC [47]. The high mutation rate found may be due to the small number of families enrolled. Giardiello et al. [48] showed an increased PDAC risk of 4.5-fold in FAP patients, in addition the association between *APC* mutations, pancreatoblastoma and neuroendocrine carcinoma was found [49,50]. The *APC* mutation found in our studyis located inexon 15 of gene (Figure 3). Neuman et al. hypothesized that mutations at the 5 ‘end of the gene could be associated with pancreatic cancers [51], however no pancreatic cancer cluster regions were reported in *APC* genes to date.

The International Cancer of the Pancreas Screening Consortium indicates the screening for PDAC onset in patients with Peutz–Jeghers syndrome, with presence of *STK11* mutation; with presence of a *CDKN2A, BRCA1/2*, or MMR mutation and with a first-degree relative affected with PDAC [52]. The American College of Gastroenterology recommends screening for individuals with mutationsin *STK11* or *CDKN2A* genes; with a mutation in *BRCA1/2, ATM, PALB2*, or MMR and a first- or second-degree relative with PDAC; with Peutz–Jeghers syndrome; and in families with at two cases of PDAC, with a first degree relative with pancreatic cancer [53].

We found no LS or FAP unaffected family members inherited the proband’s mutation, while 23 unaffected HBOC family members with mean age of 44.9 years inherited the proband’s mutation. They will undergo surveillance programs for PDAC and other related tumors developing. Currently, there are no specific guidelines on the best screening modality in mutated patients; however, the surveillance should be conducted with endoscopic ultrasound and/or magnetic resonance imaging annually, starting at age 50 [54].

The ASCO and NCCN guidelines suggested the mutational analysis of genes involved in syndromes associated with PDAC onset, not only in order to extend the mutational analysis to healthy family members who will benefit from prevention programs but must also be performed on all patients with PDAC in order to identify the carriers of mutation to direct to alternative therapies [13]. Although correlative events, *BRCA1/2* and *PALB2* mutation could have impact on treatment decisions. In this regard, Table 5 reported the current ongoing trials on the treatment of mutated patients.

Several clinical trials have shown that patients with *BRCA* mutations, treated with a platinum regimen, had longer OS than those treated with non-platinum regimen [55]. NCCN Guidelines for PDAC describe the association treatment of gemcitabina (GEM) and cisplatin (CDDP) as a possible treatment in *BRCA* mutated patients [56]. PARP inhibitors have been clinically investigated for *BRCA* mutated cases. This agent inhibits participation of PARP in base excision repair; in the presence of BRCA protein deficiency, due to mutation, this pathway is inhibited, inducing cell death [57,58]. A clinically relevant response to PARP inhibitor treatment was observed in PDAC patients reporting a mutation in *BRCA*, particularly in randomized trials on olaparib alone or veliparib in combination with cytotoxic agents [4]. It is known that immune checkpoint inhibitors have reported clinical benefits in treatment of some malignancies, such as melanoma, neuroendocrine tumors, bone malignancies, or non-small-cell lung cancer. Asthe cancer patients that reported an MMR deficiency showed response to immunotherapy, the Food and Drug Administration has approved pembrolizumab for patients with microsatellite instable tumors, such as those found in LS [59,60,61]. Moreover, a clinical trial of anti-PD-1 antibody reported a positive response in PDAC patients with MMR alterations, although further studies in a larger patient cohort are needed [17]. Of note, many novel therapies have cardiac toxicity that need to be considered [62,63,64,65]

## 5. Conclusions

Our findings showed that a high incidence of PDAC, which results in the second most recurrent tumor in HBOC and FAP families and the third in LS families, with an earlier onset and a reduced survival, could be notable in all patients with PDAC diagnosis, not only *BRCA* mutational screening, but also of other genes involved in cancer syndromes understanding PDAC. Mutation diagnosis could be useful for alternative therapeutic treatments and for prevention of related tumors onset in family members of mutated patients that inherited the mutation.

## Figures and Tables

**Table 1:** Number, tumor type, and mutations of HBOC, LS, FAP, FAMMM, and LFS probands. Mean age of onset and mutations in PDAC cases belonging to HBOC, LS, FAP, FAMMM, and LFS syndromes.

Syndrome	N° of Families (Probands Total 69)	N° and Tumor Type of Probands	Mutation Rate	N° of PDAC Cases (Total 80)	Mean Age of PDAC Onset
HBOC	56	13 pancreatic cancers; 34 breast cancers 5 ovarian cancers 3 breast and ovarian cancer 1 breast and colon cancer	(16/56) 28.6%	64	66 years
LS	7	2 pancreatic cancer 4 colon cancer 1 ovarian cancer	(2/7) 28.6%	8	58.6 years
FAP	3	1 pancreatic cancer 2 colon cancer	(1/3) 33.3%	5	48.8 years
FAMMM	2	2 melanoma	-	2	62 years
LFS	1	1 sarcoma	-	1	65 years

HBOC: Hereditary breast and ovarian cancer syndrome, LS: Lynch syndrome, FAP: Familial adenomatous polyposis, FAMMM: Familial atypical multiple mole melanoma, and LFS: Li–Fraumeni syndrome.

**Table 2:** Gender, mean age of onset, and death of PDAC patients including in HBOC, LS, and FAP mutated and non-mutated families.

	Number (77)	PDAC Patients of Non Mutated Families (55)	PDAC Patients of Mutated Families (22)	*p*-Value
Gender	Females (30)	22 (40%)	8 (36%)	0.77
	Males (47)	33 (60%)	14 (64%)	
Mean age of PDAC onset (SD)		64.5 (12.7)	62.8 (11.7)	0.59
Mean age of death (SD)		66.8 (13.3)	64.0 (11.7)	0.41

**Table 3:** Number and percentage of cancers type that occur in mutated and non-mutated HBOC families.

Type of Cancer	Cancer Number in Family	Non Mutated PDAC Patients	Mutated PDAC Patients	*p*-Value
BC	0	2 (4%)	2 (11%)	0.27
	1	15 (33%)	2 (11%)	
	2	13 (28%)	4 (22%)	
	3	10 (22%)	5 (28%)	
	≥4	6 (13%)	5 (28%)	
PDAC	1	34 (74%)	14 (78%)	0.75
	2	12 (26%)	4 (22%)	
OC	0	33 (72%)	8 (44%)	0.042
	1	11 (24%)	6 (33%)	
	2	2 (4%)	4 (22%)	
LC	0	23 (50%)	10 (56%)	0.30
	1	18 (39%)	4 (22%)	
	2	4 (9%)	3 (17%)	
	3	1 (2%)	0 (0%)	
	4	0 (0%)	1 (6%)	
LVC	0	34 (74%)	13 (72%)	0.34
	1	10 (22%)	4 (22%)	
	2	2 (4%)	0 (0%)	
	3	0 (0%)	1 (6%)	
GC	0	27 (59%)	11 (61%)	0.93
	1	15 (33%)	6 (33%)	
	2	3 (7%)	1 (6%)	
	4	1 (2%)	0 (0%)	
EC	0	33 (72%)	15 (83%)	0.71
	1	7 (15%)	1 (6%)	
	2	4 (9%)	1 (6%)	
	3	2 (4%)	1 (6%)	
CC	0	34 (74%)	14 (78%)	0.78
	1	6 (13%)	3 (17%)	
	2	4 (9%)	1 (6%)	
	3	2 (4%)	0 (0%)	
BLC	0	37 (80%)	15 (83%)	0.35
	1	8 (17%)	2 (11%)	
	2	0 (0%)	1 (6%)	
	3	1 (2%)	0 (0%)	
PC	0	40 (87%)	14 (78%)	0.62
	1	5 (11%)	3 (17%)	
	2	1 (2%)	1 (6%)	
LEU	0	41 (89%)	15 (83%)	0.53
	1	5 (11%)	3 (17%)	
bBC	0	44 (96%)	15 (83%)	0.099
	1	2 (4%)	3 (17%)	
LAC	0	39 (85%)	15 (83%)	0.77
	1	6 (13%)	3 (17%)	
	2	1 (2%)	0 (0%)	
MEL	0	43 (93%)	16 (89%)	0.54
	1	3 (7%)	2 (11%)	
KC	0	43 (93%)	17 (94%)	0.89
	1	3 (7%)	1 (6%)	
ESC	0	45 (98%)	16 (89%)	0.13
	1	1 (2%)	2 (11%)	
BRC	0	43 (93%)	17 (94%)	0.81
	1	2 (4%)	1 (6%)	
	2	1 (2%)	0 (0%)	
TC	0	42 (91%)	18 (100%)	0.20
	1	4 (9%)	0 (0%)	

BC: Breast cancer; PDAC: Pancreatic ductal adenocarcinoma; OC: Ovarian cancer; LC: Lung cancer; LVC: Liver cancer; GC: Gastric cancer; EC: Endometrial cancer; CC: Colon cancer; BLC: Bladder cancer; PC: Prostate cancer; LEU: Leukemia; bBC: Bilateral Breast cancer; LAC: Laryngeal cancer; MEL: Melanoma; KC: Kidney cancer; ESC: Esophageal cancer; BRC: Brain cancer; and TC: Thyroid cancer.

**Table 4:** Mutations identified and results of mutational analysis conducted in family members of 16HBOC mutated patients, 2LS mutated patients, and 1 FAP mutated patient. Name of mutation was reported in bold.

Family	Gene	Mutation	Exon	Family Members (Diagnosis)	Age	Mutational Analysis
1	*BRCA1*	**c.181T>G** **(p.Cys61Gly)**	5	Proband (Ovarian Cancer)	72	Mutated
				Sister (Breast Cancer)	64	Mutated
				Daughter (Unaffected)	48	Wild Type
2	*BRCA1*	**c.917delT** **(p.Leu306ArgfsTer27)**	11	Proband (Breast/Ovarian Cancer)	56	Mutated
				Son (Unaffected)	28	Mutated
				Brother (Unaffected)	61	Mutated
				Sister (Unaffected)	65	Wild Type
3	*BRCA1*	**c.5123C>A** **(p.Ala1708Glu)**	18	Proband (Pancreatic cancer)	67	Mutated
				Sister (Ovarian cancer)	63	Mutated
				Nephew (Unaffected)	38	Wild Type
				Niece (Unaffected)	40	Mutated
4	*BRCA1*	**c.5266dupC** **(p.Gln1756Profs)**	20	Proband (Breast and Ovarian cancer)	64	Mutated
				Daugheter (Unaffected)	62	Wild Type
				Son (Unaffected)	38	Mutated
				Brother (Unaffected)	66	Mutated
				Niece (Breast cancer)	32	Mutated
				Niece (Unaffected)	41	Mutated
5	*BRCA1*	**c.5266dupC** **(p.Gln1756Profs)**	20	Proband (Pancreatic Cancer)	73	Mutated
				Sister (Breast Cancer)	72	Mutated
6	*BRCA1*	**c.5397_5398del** (p.Leu1800Trpfs*29)	22	Proband (Ovarian Cancer)	63	Mutated
				Son (Unaffected)	33	Wild Type
				Daughter (Unaffected)	41	Wild Type
7	*BRCA2*	**c.3545_3546del** **(p.Gln1181_Phe1182insTer)**	11	Proband (Ovarian cancer)	57	Mutated
				Daugheter (Unaffected)	32	Wild Type
				Daugheter (Unaffected)	23	Wild Type
				Son (Unaffected)	28	Wild Type
				Brother (Unaffected)	58	Wild Type
				Sister (Ovarian cancer)	62	Mutated
				Niece (Unaffected)	34	Mutated
				Nephew (Unaffected)	30	Mutated
8	*BRCA2*	**c.4284dupT** **(p.Gln1429Serfs)**	11	Proband (Breast cancer)	85	Mutated
				Daugheter (Unaffected)	57	Wild Type
				Daugheter (Unaffected)	56	Mutated
				Son(Unaffected)	52	Wild Type
				Son(Unaffected)	50	Mutated
				Sister (Unaffected)	83	Mutated
				Niece (Unaffected)	48	Mutated
				Nephew (Unaffected)	42	Mutated
				Nephew (Unaffected)	45	Mutated
				Nephew (Unaffected)	36	Wild Type
9	*BRCA2*	**c.5511delT** **(p.Phe1837LeufsX3)**	11	Proband (Breast cancer)	46	Mutated
				Sister (Unaffected)	48	Mutated
				Sister (Unaffected)	43	Mutated
				Daughter (Unaffected)	23	Wild Type
				Daughter (Unaffected)	19	Mutated
10	*BRCA2*	**c.5722_5723delCT** **(p.L1908RfsX2)**	11	Proband (Breast cancer)	62	Mutated
				Brother (Unaffected)	61	Wild Type
11	*BRCA2*	**c.6037A>T** **(p.Lys2013Ter)**	11	Proband (Ovarian cancer)	52	Mutated
				Daugheter (Unaffected)	25	Mutated
				Son (Unaffected)	21	Wild type
				Sister (Unaffected)	58	Mutated
				Niece (Unaffected)	32	Wild type
				Niece (Unaffected)	28	Wild type
12	*BRCA2*	**c.6450dup** **(p.Val2151fs)**	11	Proband (Breast cancer)	54	Mutated
				Son (Unaffected)	24	Wild Type
				Daughter (Unaffected)	21	Mutated
13	*BRCA2*	**c.6468_6469delTC** **(p.Gln2157Ilefs)**	11	Proband (Breast Cancer)	47	Mutated
				Father (Unaffected)	75	Mutated
				Sister (Unaffected)	46	Mutated
				Sister (Unaffected)	41	Wild type
14	*BRCA2*	**c.6468_6469delTC** **(p.Gln2157Ilefs)**	11	Proband (Pancreatic Cancer)	61	Mutated
				Sister (Breast and EndometrialCancer)	62	Mutated
				Sister (Ovarian Cancer)	54	Mutated
				Nephew (Unaffected)	26	Wild type
15	*BRCA2*	**c.7857G>A** **(p.Trp2619Ter)**	17	Proband (Breast Cancer)	45	Mutated
				Sister (Unaffected)	48	Wild Type
16	*BRCA2* *BRCA2*	**c.631G>A** **(p.Val221Ile)** **c.7008-2A>T** **(IVS13-2A>T)**	7 14	Proband (Breast Cancer)	62	Mutated
				Daugheter (Unaffected)	41	Wild type
				Daugheter (Unaffected)	35	Mutated
				Brother (Unaffected)	59	Wild Type
				Sister (Bilateral Breast Cancer)	56	Mutated
				Niece (Unaffected)	35	Wild Type
				Niece (Unaffected)	30	Wild Type
				Niece (Breast cancer)	33 †	Mutated
				Cousin (Breast cancer)	70	Mutated
1	*MLH1*	**c.2181_2182delCA** **(p.Ile728Serfs)**	5	Proband (Colon Cancer)	25	Mutated
				Brother (Unaffected)	29	Wild Type
				Aunt (Breast Cancer)	58	Wild Type
				Aunt (Unaffected)	54	Wild Type
2	*MLH1*	**c.229T>C** **(p.Cys77Arg)**	3	Proband (Colon Cancer)	48	Mutated
				No family members		-
1	*APC*	**c.6709C>T** **(p.Arg2237Ter)**		Proband (Pancreatic Cancer)	52 †	Mutated
				Brother (Cerebral angiomas)	65	Mutated
				Sister (Pancreatic Cancer)	48 †	Mutated
				Sister (Colon Cancer)	55	Mutated
				Sister (Colon Cancer)	57	Mutated
				Sister (Unaffected)	67	Wild Type
				Sister (Unaffected)	66	Wild Type

†: dead.

**Table 5:** Ongoing clinical trials investigating the therapy in pancreatic ductal adenocarcinoma.

Study	Disease	Phase	ID
Niraparib in patients with germline or somatic DDRmutations	Locally advanced metastatic pancreatic cancer	Phase 2	NCT03601923
Rucaparib maintenance in patients with germline or somaticBRCA or PALB2 mutations	Locally advanced metastaticpancreatic cancer	Phase 2	NCT03140670
Olaparib in gBRCA Mutated Pancreatic Cancer Whose Disease Has Not Progressed on First Line Platinum-Based Chemotherapy	MetastaticPancreaticCancer	Phase 3	NCT02184195
Chlorambucil in Metastatic PDAC Patients Bearing a Germ Line DNA Defects Repair Mutations (SALE Trial)	MetastaticPancreaticCancer	Phase 2	NCT04692740
A Study of Maintenance Treatment withFluzoparib in gBRCA/PALB2 MutatedPancreatic Cancer Whose Disease Has Not Progressed on First Line Platinum-Based Chemotherapy	MetastaticPancreaticCancer	Phase 3	NCT04300114
Testing the Addition of Pembrolizumab, an Immunotherapy Cancer Drug to Olaparib Alone as Therapy for Patients WithPancreatic Cancer That Has Spread With Inherited BRCA Mutations	MetastaticPancreaticCancer	Phase 2	NCT04548752
Niraparib and Dostarlimab for the Treatment of Germline or Somatic BRCA1/2 and PALB2 Mutated Metastatic Pancreatic Cancer	PancreaticDuctal Adenocarcinoma	Phase 2	NCT04493060
A Randomized Study of Olaparib or Placebo in Patients With Surgically Removed Pancreatic Cancer Who Have a BRCA1, BRCA2, or PALB2 Mutation, The APOLLO Trial	P adenocarcinoma	Phase 2	NCT04858334
Niraparib and TSR-042 for the Treatment of BRCA-Mutated Unresectable or Metastatic Breast, Pancreas, Ovary, Fallopian Tube, or Primary Peritoneal Cancer	UnresectablePancreatic Carcinoma, MetastaticPancreaticCancer	Phase1	NCT04673448
Durvalumab and Olaparib for the treatment of Advanced PDAC, leiomyosarcoma or mismatch repair-proficient colorectal cancer	Advanced pancreaticcancer	Phase 2	NCT03851614

## Data Availability

Not applicable.
